# Dissertatio Medica Inauguralis de Homine dextro et sinistro

**Published:** 1783

**Authors:** 


VI. *Differtatio Medica Inauguralis de Homine dextro et finiftro. Auctore* Meinardo Simon du Pui. 4to. Lugd. Batav. 1780. p. 191.

THIS differtation relates to thofe difeafes which affect only one half of the body.

It is divided into three chapters. In the firft, which is entitled *Obfervationes morborum dimidii hominis*, the author has collected a variety of facts on this fubject, the greater number of them from books, the reft from his own obfervation. Among the latter are an inftance of jaundice affecting only the right fide of the body in an apoplectic patient, who was ftruck with hemiplegia of the fame fide; and another of a falivation confined to the right fide of the mouth, in a perfon who had made ufe of mercurial ointment to deftroy lice.

In the fecond chapter the author attempts to explain the phænomena related in the firft. Some of them he refers folely to mechanical caufes, others to the dynamic action of the veffels.

In

In the third chapter he treats of the ſuppoſed decuſſation of the nerves, and joins with Morgagni and Metzger in rejecting that doctrine.

